# Assessment of attitudes towards methadone maintenance treatment between heroin users at a compulsory detoxification centre and methadone maintenance clinic in Ningbo, China

**DOI:** 10.1186/1747-597X-8-29

**Published:** 2013-08-04

**Authors:** Yu Liu, Longhui Li, Yahai Zhang, Lina Zhang, Wenwen Shen, Huachong Xü, Guangming Wang, Weidong Lü, Wenhua Zhou

**Affiliations:** 1 Ningbo University School of Medicine, 818 Fenghua Street, Jiangbei District, Ningbo, Zhejiang 315211, China; 2 Ningbo Addiction Research and Treatment Centre, 42 Xibei Road, Haishu District, Ningbo, Zhejiang 315010, China; 3 Ningbo Municipal Public Security Bureau, 658 Zhongxing Street, Jiangdong District, Ningbo, Zhejiang 315040, China; 4 Intermediate People’s Court of Ningbo, 76 Zhongxing Street, Jiangdong District, Ningbo, Zhejiang 315040, China; 5 Laboratory of Behavioural Neuroscience, Ningbo Addiction Research and Treatment Centre, School of Medicine, Ningbo University, 42 Xibeijie St., Ningbo 315010, PR China

**Keywords:** Methadone, Compulsory detoxification centre, China

## Abstract

**Background:**

In China, the Compulsory Detoxification Centres are the main response for people who use illegal drugs. Due to high relapse rates among people released from the Compulsory Detoxification Centres, it is likely that they may seek medical help, including Methadone Maintenance Treatment (MMT) services, at some point. Therefore, better understanding of the attitudes and beliefs of people in the Compulsory Detoxification Centres can help to provide more adequate response to opioid dependence.

**Methods:**

In total, 329 detained heroin users and 112 active MMT clients were recruited from a local Compulsory Detoxification Centre and MMT clinic, respectively. The survey contained specific questions relating to attitudes and beliefs regarding MMT.

**Results:**

Participants at the Compulsory Detoxification Centre and the MMT clinic expressed different opinions, regarding positive and negative attitudes and beliefs towards MMT. In addition, participants from both sites hold certain negative attitudes and beliefs about methadone despite their acknowledgement of the positive effects of MMT. Finally, participants at the Compulsory Detoxification Centre and the MMT clinic reported distinctive treatment preferences, with the former preferring community-based treatment and the latter MMT.

**Conclusions:**

Developing targeted education about MMT for people at the Compulsory Detoxification Centres could help improve access to accurate and evidence-based health and treatment information. The study may also help providers understand and adjust services needed for target population in the future.

## Introduction

The inclusion of MMT as part of opioid dependence treatment system in China remained controversial for a long period of time [1]. Methadone had been available only for the purpose of acute detoxification in China between 1993 and the early 2000s [1-3]. Recognizing the success of MMT worldwide and the alarming rates of HIV cases among Chinese injection drug users [4-6], there has been a rapid expansion of MMT services in the country. Currently, there are more than 600 MMT clinics and more than 175,000 patients have received MMT in China [7]. The number of registered illicit drug users, mainly heroin users, has increased from 70,000 in 1990 to 1,336,000 in 2009. Thus, MMT programs have been widely accepted in China by authorities as an effective therapeutic approach to treat opioid dependence and a prevention strategy to reduce the transmission of various infection diseases [8]. It is worth noting that MMT is currently the most available opioid substitution treatment (OST) service in China, although OST is not limited to MMT.

The goal of MMT is to reduce and even eliminate heroin use by stabilizing patients on methadone for as long as is necessary and to help them avoid returning to previous patterns of drug use and change in risk behaviours, particularly injecting drugs [9-11]. The retention rates of MMT clinics have been suggested to be complicated by a number of factors. Various pre- and in-treatment predictors (i.e. marital status, employment, gender, methadone dosage) have been shown internationally to influence clients’ decision to enter treatment, medical compliance and treatment outcomes [12-15]. In addition to clients’ characteristics and the quality of MMT services, a significant portion of MMT clients in other contexts have also reported negative attitudes towards methadone and expressed a strong desire to discontinue methadone treatment as soon as possible [16-20]. Common negative attitudes about MMT include the beliefs that methadone is addictive, that it is more difficult to withdrawal from methadone than heroin, and that methadone is bad for health [17,21-23]. A wealth of literature has documented that patients’ attitudes on treatment can substantially affect the client-provider therapeutic alliance and treatment duration [24-26]. Indeed, negative perceptions towards OST have been found to adversely affect treatment outcomes, enrollment and retention rates among former or current injection drug users [15,21,22].

According to latest China Anti-drug Law, drug addicts are subjective to compulsory detoxification for up to two years, only after being convicted to violate the regulations of community treatment. Reeducation through labor is no long applied to drug addicts and the isolated compulsory detoxification centers would be the only authorized agency. Based on regular assessments carried out in the Compulsory Detoxification Centres, drug addicts can be released ahead of time or detention can also be prolonged for one more year. A number of surveys have consistently reported extremely high rates of relapse among people recently released from the Compulsory Detoxification Centres [27-29]. With relapse rates among people leaving the Compulsory Detoxification Centres as high as above 90% within a year [27-29], it is likely that released people may seek medical help, including MMT services, at some point. Therefore, to understand and address the attitudes of detainees at the Compulsory Detoxification Centres towards MMT would be of great importance to help better understand their needs on concerns about OST. Released drug addicts from the detoxification centers are subjected to continuous rehabilitation in their local residential communities for up to three years. The communities addressed in our Anti-drug law represent an open environment where the recovering drug users normally resident. The provision of effective and timely medical services to people who use drugs in the community-based treatment system is critical to achieving the explicit long-term goals of the government’s current treatment programs, abstinence and recovery.

As mentioned previously, extremely high rates of relapse are reported among newly released drug users. It is of importance for the newly released drug users to use methadone when necessary. Most clinical studies conducted in China have been focused on active MMT clients and the effect of treatment-related factors on clinic retention rates. It should be noted that the majority of detained heroin users have been in and out of the Compulsory Detox Centre more than once and they also experienced methadone treatment at some point. However, there is a paucity of reports that have investigated the attitudes and concerns of detainees at the Compulsory Detoxification Centres towards methadone. The primary goal of the current study is to explore the attitudes on MMT between participants’ in a local Compulsory Detoxification Centre and a MMT clinic. Furthermore, the study also examines treatment preferences and source of MMT information among participants at the Compulsory Detoxification Centre and MMT clinic. It was hypothesized that the detained heroin users held different opinions towards MMT, compared with those receiving MMT at the local clinics.

## Methods

### Participants

A brief self-administered, written survey was carried out at Ningbo Methadone Clinical Centre and Ningbo Compulsory Detoxification Centre. In total, 329 patients with the history of heroin use were recruited from the centre. Ningbo Compulsory Detoxification Centre was operated by local law enforcement. During compulsory detoxification, pharmacological treatment is allowed to be prescribed to mainly ease physiological discomfort caused by withdrawal. Psychological treatment and counseling is also available. At Ningbo MMT Clinic, a total of 112 MMT clients participated in the study. The clinic was operated by local department of health. All participants were required to have been in methadone maintenance for at least three months continuously prior to participation in the study. Patients meeting the criteria filled out the survey while at the clinic. In the current study, the research staff ensured that participants understood the decision to participate was voluntary and refusal would not have negative repercussions. Before the survey was administered, eligible participants received consent forms distributed by research staff. Anonymity in the data collection process was ensured by not soliciting names or other identifying details on the questionnaire. While staff working at the Compulsory Detoxification Centre helped distribute and collect the surveys to detainees at the centre, detainees completed the survey without the presence of the centre staff. Basic demographic characteristics, including gender, age, ethnic groups, employment (before being arrested), marital status, and heroin use history were collected as part of the survey. The present study was approved by the Institutional Review Board of Ningbo Addiction Research and Treatment Centre.

### Questionnaire

The survey contained specific questions relating to a wide range of attitudes (agree, don’t know, and disagree) regarding MMT. The survey mainly assessed the participants’ attitudes towards MMT itself and current MMT services, which were divided into “positive” and “negative” attitudes. The evaluation of “positive” attitudes towards MMT included “MMT helps me live a normal life”, “MMT can help decrease craving”, “MMT can help reduce the consumption of illicit drugs”, and “MMT can help prevent HIV infection”. The “negative” attitudes towards MMT included “methadone is addictive”, “it is more difficult to stop using methadone than other opioids”, “I would be looked down upon by non-methadone treated patients”, “methadone is bad for my health” and “my family members would feel shameful about if I was treated with methadone”. The evaluation component includes the following items: source of MMT information, treatment preference, and reasons for not choosing MMT as their preferred treatment.

### Data analysis

Descriptive analyses were performed to describe the demographic and attitude variables of participants. A comparison was carried out between the two groups in socio-demographic data and drug use related characteristics. The prevalence of specific knowledge and attitudes were determined between two populations. All survey responses were transformed into categorical variables. Pearson *χ*2 was used to examine the differences of demographics and drug use related characteristics and specific attitudes (agree, don’t know, disagree) between two groups. All t-tests were two-sided and p-value less than 0.05 were considered statistically significant. All statistical analyses were performed using SPSS 10.

## Results

### Demographics of participants

Participants’ basic demographics and heroin use history are summarized in Table 1. Participants averaged 32.4 (± 7.2) and 35.0 (± 6.3) years of age from the Detoxification Centre and MMT Clinic, respectively. The majority of participants were male (Detoxification: 77.5%; MMT: 75.9%) and Han Chinese (Compulsory Detoxification: 97.9%; MMT: 99.0%) at both sites. The majority of participants at the Compulsory Detoxification Centre and MMT clinic are either single (Detoxification: 47.1%; MMT: 42.0%) or married (Compulsory Detoxification: 39.2%; MMT: 33.1%). In contrast, only a small portion of participants at both sites were either divorced (Compulsory Detoxification: 11.9%; MMT: 12.5%) or windowed (Compulsory Detoxification: 2%; MMT: 0%). A large percentage of participants at the Compulsory Detoxification Centre were unemployed before they had been arrested (70.2%). The majority of MMT clients indicated that they are currently unemployed (57.1%), with self-employment as the most common form of employment. One third of participants at the Compulsory Detoxification Centre self-reported daily using heroin (31.9%) before having been arrested, which was similar to participants who frequently (defined as 1-3 times a week) (35.9%) or occasionally (defined as less than once a week) used heroin (32.2%). In contrast, almost half of active MMT patients were daily heroin users, compared with frequent (28.6%) or occasional (12.5%) heroin users prior to the enrollment of MMT programmes. The following characteristics are found to be significantly different between two groups: age (*p* < 0.01), prevalence of daily, frequent and occasional use of heroin (*p* < 0.05).

**Table 1:** Basic demographic characteristics of compulsory detoxification and MMT patients

	**Compulsory detoxification**	**MMT**
**Number of participants**	329	112
**Male**	255 (77.5%)	85 (75.9%)
**Female**	67 (20.4%)	18 (16.1%)
**Age**	16–50	19–51
**Male**	16–50	19–51
**Female**	21–44	27–45
**Ethnicity**		
**Han**	322 (97.9%)	96 (85.7%)
**Minority**	5 (1.5%)	1 (1%)
**Marital Status**		
**Single**	155 (47.1%)	47 (42.0%)
**Married**	129 (39.2%)	37 (33.1%)
**Divorced**	39 (11.9%)	14 (12.5%)
**Widowed**	2 (0.6%)	0 (0.0%)
**Employment**		
**Full-time**	38 (11.6%)	12 (10.7%)
**Part-time**	52 (15.8%)	16 (14.3%)
**Unemployed**	231(70.2%)	64 (57.1%)
**Heroin Use**		
**Daily**	105 (31.9%)	54 (48.2%)
**Frequently**	118 (35.9%)	32 (28.6%)
**Occasionally**	106 (32.2%)	14 (12.5%)
**Never**	0 (0.0%)	2 (1.8%)

### Positive attitudes towards MMT

**Figure 1:**
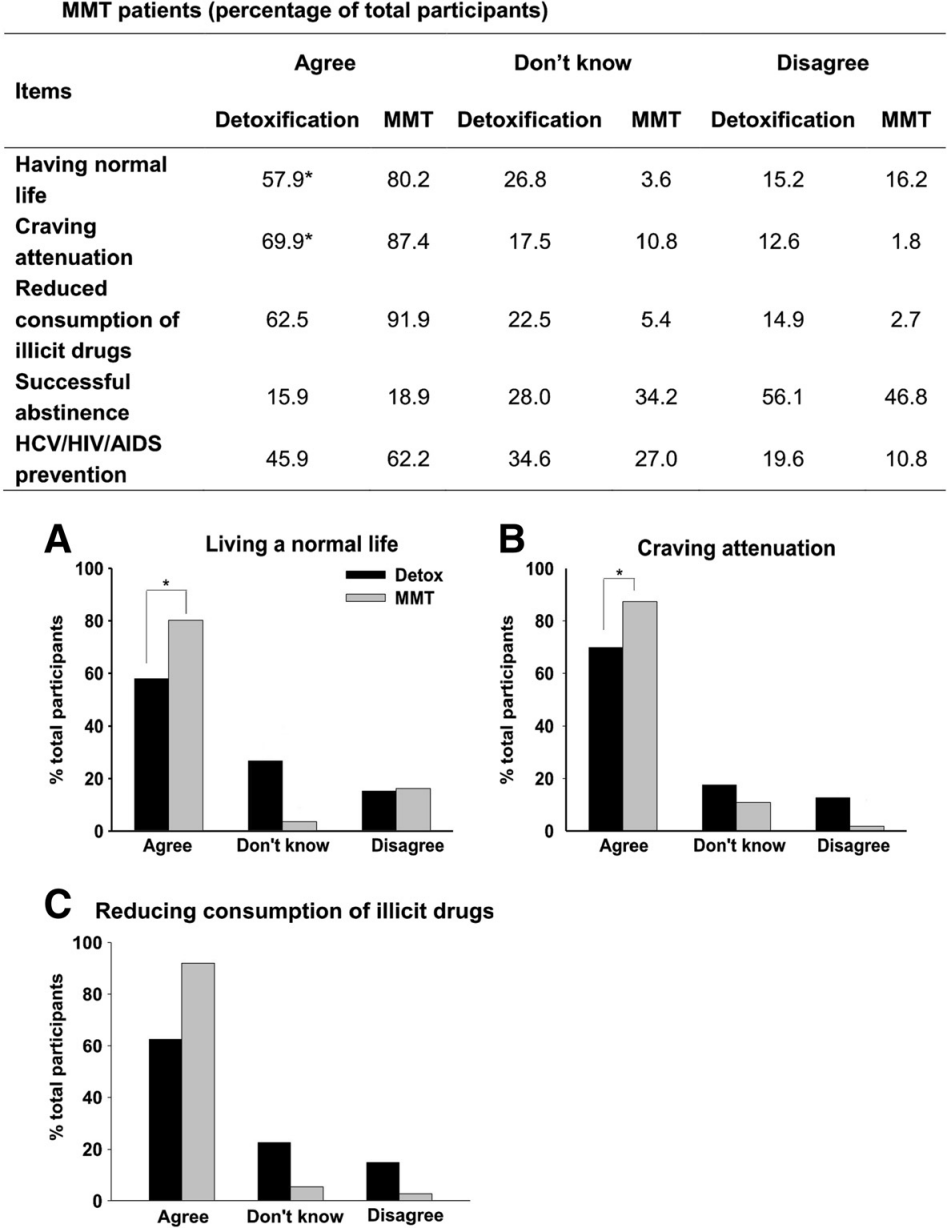
**Positive attitudes toward MMT between participants at the Compulsory Detoxification Centre (black bars) and MMT patients (grey bars).** Points represent the percentage of total participants agreed, didn’t know or disagreed each item of the questionnaire. **(A)**: Methadone help me live a normal life; **(B)**: Methadone help attenuate craving; **(C)**: Methadone help reduce the consumption of illicit drugs. Asterisk (*) denotes a significant difference between Compulsory Detoxification and MMT patients, p < 0.05.

Participants’ responses to the “positive” attitudes towards MMT are summarized in Figure 1. A majority of MMT patients reported “positive” attitudes towards MMT, including believing that entering the MMT programme could help them with “living a normal life” (80.2%), “craving attenuation” (87.4%), “reducing illegal drug consumption (91.9%), and “preventing HCV and HIV/AIDS” (62.2%). In contrast, a lower percentage of participants at the Compulsory Detoxification Centre believed that MMT could help them with “living a normal life” (57.9%), “craving attenuation” (69.9%), “reducing consumption of illegal drugs (62.5%), “preventing HCV/HIV/AIDS” (45.9%).

### Negative attitudes towards MMT

**Figure 2:**
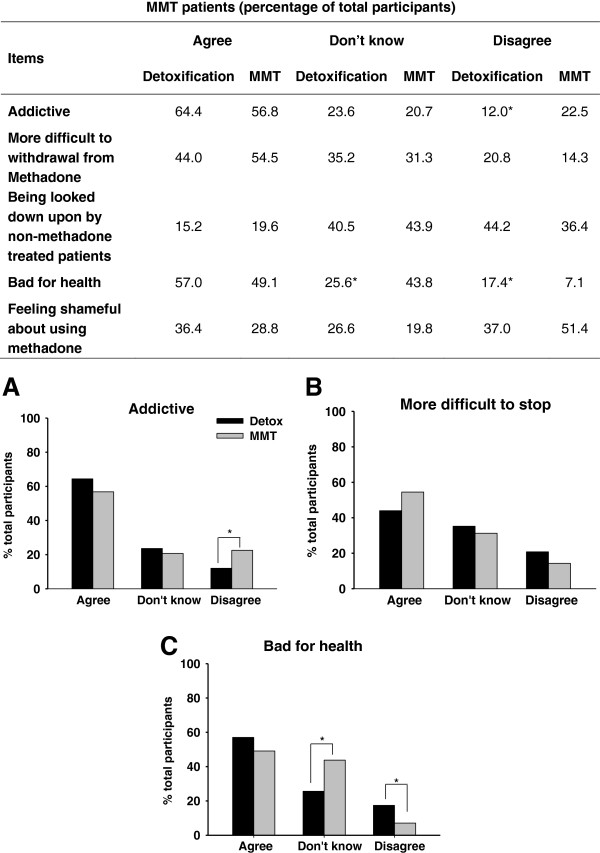
**Negative attitudes toward MMT between participants at the Compulsory Detoxification Centre (black bars) and MMT patients (grey bars).** Points represent the percentage of total participants agreed, didn’t know or disagreed each item of the questionnaire under “negative attitude” category. **(A)**: Methadone is addictive **(B)**: it is more difficult to stop using methadone; **(C)**: Methadone is bad for health. Asterisk (*) denotes a significant difference between Compulsory Detoxification and MMT patients, p < 0.05.

Participants’ negative attitudes towards MMT were summarized in Figure 2. Participants at the Compulsory Detoxification Centre and the MMT clinic reported negative attitudes towards MMT in a similar manner. That is, a large percentage of the participants, regardless of treatment approaches, agreed that “Methadone is addictive” (Compulsory Detoxification: 64.4%; MMT: 56.8%), “Methadone is more difficult to stop using” (Compulsory Detoxification: 36.4%; MMT: 53.0%), “MMT patients would be looked down upon by non-methadone maintained patients” (Compulsory Detoxification: 15.2%; MMT:19.6%), “Methadone is bad for health” (Compulsory Detoxification: 44.0%; MMT: 54.5%). In addition, approximately one third of participants from both sites reported that “family members would feel shame if they knew I was in MMT” (Compulsory Detoxification, 36.4%; MMT: 28.8%).

### Sources of MMT information and treatment preference

**Figure 3:**
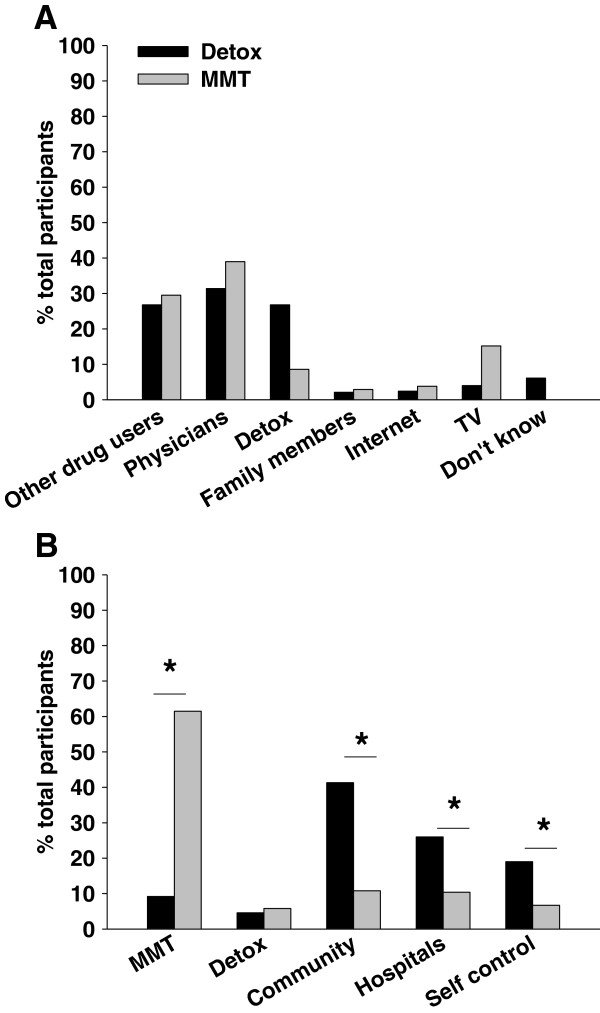
**Sources of MMT information and treatment preference among participants at the Compulsory Detoxification Centre and MMT patients.(A)** sources of MMT information; **(B)** Treatment preferences. Asterisk (*) denotes a significant difference between Compulsory Detoxification and MMT patients, p < 0.05.

Patients reported their initial sources of MMT information and treatment preferences in the survey and these results are summarized in Figure 3. “Other people who use drugs” (Compulsory Detoxification: 26.8%; MMT: 29.5%) and physicians (Compulsory Detoxification: 31.4%; MMT: 39.0%) were both common initial sources of information about MMT for participants. In addition, the Compulsory Detoxification Centre was the initial source of MMT information for 26.8% of the detainees; whereas TV was the initial source for 15.2% of the interviewed MMT patients. When asked to indicate their “most preferred” treatment, the most common response of detainees at the Compulsory Detoxification Centre was “community treatment” (41.3%), followed by inpatient treatment (26.0%), “self-detoxification” (19.0%), and then MMT (6.8%). In contrast, when given the same choices, almost two thirds of MMT patients preferred MMT (61.5%), followed by inpatient treatment (10.4%) and community treatment (8.6%). Compulsory Detoxification (10.8%) and self Detoxification (6.7%) were less popular choices. When asked their biggest concern to enter MMT programme, MMT patients reported “methadone is more difficult to stop using than other opioids” (41.7%), followed by “methadone is more addictive than other opioids”(19.4%), “receiving treatment at MMT clinics is inconvenient” (11.2%), and finally “methadone is bad for health” (3.7%).

## Discussion

The current study was aimed to explore potential barrier to long-term MMT treatment among detainees at a local Compulsory Detoxification Centre by identifying their attitudes and beliefs towards MMT and comparing with those of active MMT patients. The major findings are: (1) detainees at the Compulsory Detoxification Centre expressed less positive and more negative attitudes and beliefs about MMT when compared to active MMT patients; (2) participants from both sites showed rather negative attitudes towards methadone; (3) detainees at the Compulsory Detoxification Centre and MMT patients reported distinctive treatment preferences, with the former indicating a much higher interest in community treatment and much lower interest in MMT as their preferred treatment choice than the latter.

Studies have demonstrated that in-treatment patients had significantly more positive attitudes toward MMT than the out-of-treatment group [21,22,30]. In line with these findings, our study also showed that a lower percentage of detainees at the Compulsory Detoxification Centre reported positive attitudes towards MMT, compared with active MMT clients. These differences highlight the fact that a potentially important obstacle to continue MMT among released people from the Compulsory Detoxification Centres, when necessary, would be their negative attitudes towards MMT. The results of this study indicate that over 90% of detainees who will soon be leaving Compulsory Detoxification Centre do not list MMT as their preferred method of treatment. In this context, it is needed to develop a targeted and suitable education programs about MMT in the setting of Compulsory Detoxification Centre. Since patients’ views on treatment can greatly influence their medical compliance and treatment outcomes, how to decrease stigma surrounding methadone and better handle patients’ potential concerns with the medications among in- and out-of-treatment patients should be one of the primary goals for the education programs.

Another interesting finding of this study was that detainees at the Compulsory Detoxification Centre and MMT patients reported different preferences for treatment options. Community-based treatment and inpatient treatment were frequently chosen as the most desirable treatment for detainees at Compulsory Detoxification Centre. In contrast, the majority of active MMT patients prefer OST. Given the attitude differences between in- and out-of-treatment patients, this also confirms the notion that actual experiences of MMT therapy could profoundly shape the patients’ opinions on various aspects of MMT. On the other hand, these findings may represent a trend that the MMT patients are self-selecting and the patients who like MMT are already in the treatment. Over one third of the detainees at the Compulsory Detoxification Centre indicated that they preferred “community-based treatment”, the new component of China New Drug Control Law [31]. It should be noted that community-based treatment/recover in China is often defined as patients living in an open residential area under the supervision of the sub-district officials of these communities. This model greatly differs from the more commonly accepted community-based treatment system which involves a highly structured and somewhat isolated services [31]. However, full details of such a treatment system are lacking in the New Drug Control Law and the implementation of community-based treatment is at the stage of exploration across the nation. There is great demand for community treatment/recovery among detainees at Compulsory Detoxification Centre. If the community-treatment/recovery programs are operated efficiently, these programs may substantially increase the chances of released people to recover.

Previous studies have shown that even high-dose methadone is not effective for a subgroup of opioid users which accounts for 15-20% of patients with a diagnosis of opioid dependence [32]. A wealth literature has documented the effectiveness of OST/MMT, regarding various risky and negative consequences associated with opioid dependence [9-11]. Indeed, MMT has been widely accepted by the authority as a “first-line” treatment for opioid users in China. At the moment, China has limited capacity to deliver both psychological and drug treatment services outside of methadone clinics and the hospital system. The authors believe that individualized treatment, based on the patients’ preference and conditions, should be developed, including buprenorphine maintenance, counseling, narcotic association etc. To truly serve the diverse needs of a large number of patients who come out of the Compulsory Detoxification Centres, more robust and personalized services should be developed under the label of “community-based treatment/recovery”.

There are several limitations of this study’s methodology that need to be addressed here. Generalization of these results must be made with caution as Ningbo’s drug treatment services may not be representative of other Compulsory Detoxification Centre and MMT clinics in China and worldwide. In addition, the number of patients at the Compulsory Detoxification Centre who had used MMT before was not assessed in the current study. It is likely that detainees with MMT experiences at the Compulsory Detoxification Centre may view methadone differently from those who had not received MMT. It is either due to the fact that active MMT patients accept MMT as a desirable concept in the first place or their experiences in treatment helped to change their attitudes. To the best of our knowledge, the majority of detained heroin users have were in and out of the compulsory detoxification centre more than once and they experienced methadone treatment at some point. Therefore, the participants from the compulsory detox centre could still provide insights about their attitudes towards methadone. The level of opioid dependence and history of heroin of the participants was not obtained in the current study. The prevalence of previous heroin use (daily, frequent and occasional use) was also significantly different between two populations. These can be the confounding factors for the differences in the attitudes towards MMT between two groups. Furthermore, at the MMT clinic, the patients participated in the study voluntarily. In contrast, due to the regulations of the Compulsory Detoxification Centre, the centre staff, instead of the researchers, distributed and collected the surveys. The different interview processes may have influenced the results as participants’ attitudes and beliefs about MMT. Finally, it is worth noting that there have been ethical concerns of conducting research in the setting of Compulsory Detoxification Centres in China 29,33,34. In the current study, the ethical issues and objectiveness of the results were considered and addressed. For example, all the participants signed the informed consent and detainees completed the survey without the presence of the centre staff.

## Conclusions

The prevention of released people who use drugs from relapsing is the most important issue in the country’s response to drug treatment. The incarcerated population recruited from a compulsory detox centre is candidate for methadone maintenance treatment after being released from the centre. As a matter of fact, detained drug users are strongly encouraged to use methadone treatment, since the relapse rates are particularly high among newly released drug users. The current study has identified negative attitudes about MMT among the detainees at the Compulsory Detoxification Centre. Developing more targeted education about MMT for this given population, improving the quality of existing methadone treatment, and expanding the treatment options available to those exiting Compulsory Detoxification Centres would be essential to increase the efficacy of Chinese MMT for opiate dependency.

## Abbreviations

MMT: Methadone maintenance treatment; OST: Opioid substitution treatment.

## Competing interests

The authors declare that they have no competing interests.

## Authors’ contributions

YL and WZ designed the study. YL, LL, YZ, HX, GW and WL conducted the research. LL, YZ, LZ and WS conducted the data analysis. YL and WZ prepared the manuscript. All authors read and approved the final manuscript.

## References

[B1] Tang YL Zhao D Zhao C Cubells JF Opiate addiction in China: current situation and treatments Addiction 2006 101 5 657 665 10.1111/j.1360-0443.2006.01367.x 16669899

[B2] Duan L-X Xu G-Z A clinical observation on detoxification of heroin dependence by oral administration of methadone Chinese Journal of Drug Abuse Prevention and Treatment 2003 9 6 8

[B3] Lu L Zhao D Bao YP Shi J Methadone maintenance treatment of heroin abuse in China Am J Drug Alcohol Abuse 2008 34 2 127 131 10.1080/00952990701876989 18293229

[B4] Bruce RD Methadone as HIV prevention: high volume methadone sites to decrease HIV incidence rates in resource limited settings Int J Drug Policy 2010 21 2 122 124 10.1016/j.drugpo.2009.10.004 19931444 PMC2839048

[B5] Degenhardt L Mathers B Vickerman P Rhodes T Latkin C Hickman M Prevention of HIV infection for people who inject drugs: why individual, structural, and combination approaches are needed Lancet 2010 376 9737 285 301 10.1016/S0140-6736(10)60742-8 20650522

[B6] Mathers BM Degenhardt L Ali H HIV prevention, treatment, and care services for people who inject drugs: a systematic review of global, regional, and national coverage Lancet 2010 375 9719 1014 1028 10.1016/S0140-6736(10)60232-2 20189638

[B7] Sullivan SG Wu Z Rapid scale up of harm reduction in China Int J Drug Policy 2007 18 2 118 128 10.1016/j.drugpo.2006.11.014 17689354

[B8] Rou K-M Wu Z-Y Promoting universal access of AIDS Programmemes for drug using population in China Chinese Journal of Drug Dependence 2009 18 3 172 174

[B9] Connock M Juarez-Garcia A Jowett S Methadone and buprenorphine for the management of opioid dependence: a systematic review and economic evaluation Health Technol Assess 2007 11 9 1 171 iii-iv 17313907 10.3310/hta11090

[B10] Larney S Does opioid substitution treatment in prisons reduce injecting-related HIV risk behaviours? A systematic review. Addiction 2010 105 2 216 223 10.1111/j.1360-0443.2009.02826.x 20078480

[B11] Tross S Hanner J Hu MC Pavlicova M Campbell A Nunes EV Substance use and high risk sexual behaviors among women in psychosocial outpatient and methadone maintenance treatment programs Am J Drug Alcohol Abuse 2009 35 5 368 374 10.1080/00952990903108256 20180666 PMC2846384

[B12] del Rio M Mino A Perneger TV Predictors of patient retention in a newly established methadone maintenance treatment programme Addiction 1997 92 10 1353 1360 10.1111/j.1360-0443.1997.tb02854.x 9489052

[B13] Magura S Nwakeze PC Demsky SY Pre- and in-treatment predictors of retention in methadone treatment using survival analysis Addiction 1998 93 1 51 60 10.1046/j.1360-0443.1998.931516.x 9624711

[B14] Steer RA Psychosocial correlates of retention in methadone maintenance Int J Addict 1980 15 7 1003 1009 7450944 10.3109/10826088009040074

[B15] Bobrova N Alcorn R Rhodes T Rughnikov I Neifeld E Power R Injection drug users' perceptions of drug treatment services and attitudes toward substitution therapy: a qualitative study in three Russian cities J Subst Abuse Treat 2007 33 4 373 378 10.1016/j.jsat.2007.02.002 17499960

[B16] Goldsmith DS Hunt DE Lipton DS Strug DL Methadone folklore: beliefs about side effects and their impact on treatment Hum Organ 1984 43 4 330 340

[B17] Hunt DE Lipton DS Goldsmith DS Strug DL Spunt B "It takes your heart": the image of methadone maintenance in the addict world and its effect on recruitment into treatment Int J Addict 1985 20 11–12 1751 1771 3833809 10.3109/10826088509047261

[B18] Stancliff S Myers JE Steiner S Drucker E Beliefs about methadone in an inner-city methadone clinic J Urban Health 2002 79 4 571 578 10.1093/jurban/79.4.571 12468676 PMC3456722

[B19] Zule WA Desmond DP Attitudes toward methadone maintenance: implications for HIV prevention J Psychoactive Drugs 1998 30 1 89 97 10.1080/02791072.1998.10399674 9565212

[B20] Zweben JE Payte JT Methadone maintenance in the treatment of opioid dependence. A current perspective West J Med 1990 152 5 588 599 2190427 PMC1002416

[B21] Peterson JA Schwartz RP Mitchell SG Why don't out-of-treatment individuals enter methadone treatment programmes? Int J Drug Policy 2010 21 1 36 42 10.1016/j.drugpo.2008.07.004 18805686 PMC2790538

[B22] Zaller ND Bazazi AR Velazquez L Rich JD Attitudes toward methadone among out-of-treatment minority injection drug users: implications for health disparities Int J Environ Res Public Health 2009 6 2 787 797 10.3390/ijerph6020787 19440415 PMC2672350

[B23] Zweben JE Sorensen JL Misunderstandings about methadone J Psychoactive Drugs 1988 20 3 275 281 10.1080/02791072.1988.10472498 3069986

[B24] Broome KM Knight DK Knight K Hiller ML Simpson DD Peer, family, and motivational influences on drug treatment process and recidivism for probationers J Clin Psychol 1997 53 4 387 397 10.1002/(SICI)1097-4679(199706)53:4<387::AID-JCLP12>3.0.CO;2-C 9169394

[B25] Joe GW Simpson DD Dansereau DF Rowan-Szal GA Relationships between counseling rapport and drug abuse treatment outcomes Psychiatric Servercies 2001 52 9 1223 1229 10.1176/appi.ps.52.9.1223 11533397

[B26] Kasarabada ND Hser YI Boles SM Huang YC Do patients' perceptions of their counselors influence outcomes of drug treatment? J Subst Abuse Treat 2002 23 4 327 334 10.1016/S0740-5472(02)00276-3 12495794

[B27] Zhu WX Dong JQ Hesketh T Preventing relapse in incarcerated drug users in Yunnan Province, China Drug Alcohol Rev 2009 28 6 641 647 10.1111/j.1465-3362.2009.00068.x 19930018

[B28] Zhao C Liu Z Zhao D Liu Y Liang J Tang Y Liu Z Zheng J Drug abuse in China Ann N Y Acad Sci 2004 1025 439 445 10.1196/annals.1316.054 15542747

[B29] Cohen JE Amon JJ Health and human rights concerns of drug users in detention in Guangxi Province, China PLoS Med 2008 5 12 e234 10.1371/journal.pmed.0050234 19071954 PMC2596857

[B30] Schwartz RP Kelly SM O'Grady KE Attitudes toward buprenorphine and methadone among opioid-dependent individuals Am J Addict 2008 17 5 396 401 10.1080/10550490802268835 18770082 PMC2814176

[B31] Liu Y Liang J Zhao C Zhou W Looking for a solution for drug addiction in China: exploring the challenges and opportunities in the way of China's new drug control law Int J Drug Policy 2010 21 3 149 154 10.1016/j.drugpo.2009.10.002 19896818

[B32] Jürgens R Csete J Amon JJ Baral S Beyrer C People who use drugs, HIV, and human rights Lancet 2010 376 9739 475 485 10.1016/S0140-6736(10)60830-6 20650514

